# Adaptive deep Q-networks for accurate electric vehicle range estimation

**DOI:** 10.3389/fdata.2025.1697478

**Published:** 2025-11-27

**Authors:** Urvashi Khekare, Rajay Vedaraj I. S.

**Affiliations:** 1 School of Mechanical Engineering, Vellore Institute of Technology, Vellore, Tamil Nadu, India; 2 School of Computer Science and Engineering, Vellore Institute of Technology, Vellore, Tamil Nadu, India

**Keywords:** driving range, state of charge, electric vehicle parameters, deep reinforcement learning, pathfinder optimization, fuzzy k-means algorithm

## Abstract

It is critical that electric vehicles estimate the remaining driving range after charging, as this has direct implications for drivers' range anxiety and thus for large-scale EV adoption. Traditional approaches to predicting range using machine learning rely heavily on large amounts of vehicle-specific data and therefore are not scalable or adaptable. In this paper, a deep reinforcement learning framework is proposed, utilizing big data from 103 different EV models from 31 different manufacturers. This dataset combines several operational variables (state of charge, voltage, current, temperature, vehicle speed, and discharge characteristics) that reflect highly dynamic driving states. Some outliers in this heterogeneous data were reduced through a hybrid fuzzy k-means clustering approach, enhancing the quality of the data used in training. Secondly, a pathfinder meta-heuristics approach has been applied to optimize the reward function of the deep Q-learning algorithm, and thus accelerate convergence and improve accuracy. Experimental validation reveals that the proposed framework halves the range error to [−0.28, 0.40] for independent testing and [−0.23, 0.34] at 10-fold cross-validation. The proposed approach outperforms traditional machine learning and transformer-based approaches in Mean Absolute Error (outperforming by 61.86% and 4.86%, respectively) and in Root Mean Square Error (outperforming by 6.36% and 3.56%, respectively). This highlights the robustness of the proposed framework under complex, dynamic EV data and its ability to enable scalable intelligent range prediction, which engenders innovation in infrastructure and climate conscious mobility.

## 1 Introduction

Accurately predicting the electric vehicle range is quite a big challenge due to large variations in both battery dynamics and driving conditions. In most model applications of range prediction through machine learning, there are difficulties regarding dynamic adaptability and generalization across different types of vehicles (Kumar and Alok, 2020; Sanguesa et al., 2021). Electric vehicles are widely promoted as an environmentally friendly transportation option, but their actual environmental impact strongly depends on the energy mix used for charging. Electricity from renewable sources such as solar, wind, or hydro power can lead to significant reductions in greenhouse gas emissions and air pollutants when used by EVs. However, in regions where electricity generation still relies on fossil fuels like coal or oil, the overall lifecycle emissions of EVs may approach or even exceed those of efficient internal combustion vehicles. Therefore, assessing EV sustainability requires consideration of both battery performance and the carbon intensity of the local power grid. However, a major complication that arose in the development of electric vehicles is the power battery. To enhance the performance of the battery in each driving experience, the electric vehicle is equipped with a lithium-ion battery (Tian et al., 2020). The battery offers a long lifespan, high energy density (Rallo et al., 2020), and the ability to provide high energy density (Pande and Khekare, 2024; Khan et al., 2023). The batteries used in electric vehicles are complex since they tend to experience problems such as battery degradation (Mahmud et al., 2023), self-discharge, unmanaged energy usage, by using energy to operate the entire vehicle to be operated, and thermal runaway (Chavan et al., 2023; Yao et al., 2021; Dhote et al., 2023). The state of health (Wu et al., 2022) and state of charge (Kim and Chung, 2023) are the parameters that reflect the capacity of the battery to some extent. Due to the internal reactions of the battery, the state of health estimation is not accurate (Nuroldayeva et al., 2023). The uncontrolled and prolonged distribution of thermal effect and load current causes the battery to suffer from inconsistency, which directly influences the state of charge (SoC) of the battery, terminal voltage and output energy.

### 1.1 Background

Directly related to increased electric vehicle adoption, energy demand and the logistics of electricity supply for transport are highly dependent factors. With the increase in the number of EVs, the overall power demand rises, putting additional loads on the grid without disturbing grid stability. Environmental outcomes greatly depend on grid emission intensity, reflecting the share of renewable and fossil-based electricity generation. Charging patterns, like the time, frequency, and rate at which a vehicle is charged, also fall into a very important category because off-peak or renewable-supported charging can reduce emissions greatly. Combining renewable integration with smart charging has been demonstrated by research studies, such as (Kumar Jha et al., 2025), as an effective way to bring about drastic cuts in energy demand and greenhouse gas emissions for different EV adoption scenarios. Hence, proper EV performance evaluation needs accurate estimation of both the carbon intensity of the generation mix and the temporal dynamics of charging behavior. The complications in the process mainly arise with difficulties in estimating the health status of the vehicle and its parameters describing battery degradation, which makes the estimation of the distance it has traveled with available energy quite difficult. Driving range prediction is actually this process, which is of vital importance. Incorrect predictions can make the driver anxious because the uncertainty of the remaining driving distance might affect the whole journey. A multi-level granularity fragments-based machine learning algorithm is used here to assess the energy utilization in every driving experience. The data are classified into trip fragments, micro-fragments, and kinematic fragments. Based on these fragments, the energy consumption is predicted. A linear regression-based long short-term memory is used to determine the range of electric vehicles concerning the state of charge; when the dataset is imbalanced, the prediction accuracy drops significantly (Zhang et al., 2020).

The electric vehicle range is predicted by taking into consideration state of charge, voltage, and energy level using a gradient boost decision tree algorithm (Sun et al., 2019). The method followed requires a large-scale dataset to enhance the accuracy of the prediction. Energy consumption in an electric vehicle will also be affected by atmospheric effects; therefore, different types of electric vehicles will have to be analyzed under different climatic conditions to verify their driving range. In this analysis, the average energy consumed by the vehicle is affected by an increase in temperature (Hao et al., 2020). Parameters of the vehicle. For parameters related to speed and the braking process, the driving range is predicted with the use of microsimulation to verify the prediction algorithms. In improving the range of electric vehicles, literature has adopted a control model even though there are problems in existing methods for accurately estimating the dynamic vehicle's average range. The proposed model is analytical, which complicates designing the vehicle parameters.

Electric vehicles play a vital role in global decarbonization and sustainable mobility strategies. However, one of the key challenges that impedes wider diffusion of EVs is the uncertainty related to driving range estimation under varying environmental and operational conditions. This not only causes “range anxiety” among users but also complicates fleet management, route planning, and energy optimization in smart grids. Traditional approaches, including rule-based models, regression, and standard machine learning, fall well short in their ability to capture the dynamic non-linear relationships that exist between the status of the battery, vehicle parameters, and driving behavior. The majority of them require extensive labeled datasets for each configuration of the vehicle, are non-adaptable in real time, or fail to generalize across diverse EV profiles. The efficiency of electric traction systems has been shown to outperform that of conventional fuel-based transport due to lower energy losses and higher conversion performance (Skrúcaný et al., 2018). Moreover, recent research highlights that optimizing vehicle aerodynamics can further enhance energy efficiency and extend the driving range, particularly in sport utility vehicles (Quan, 2024).

**Figure 1:**
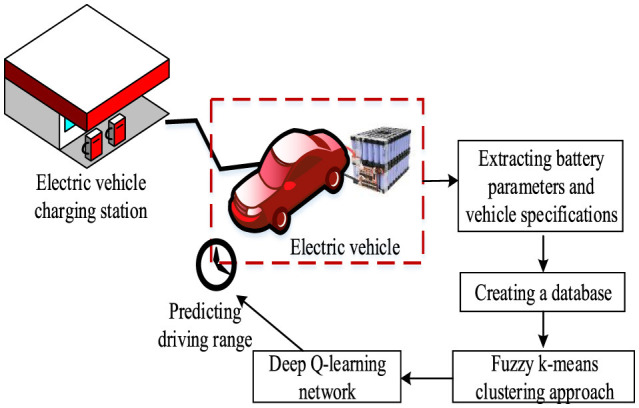
Proposed driving range prediction model.

The other recent range prediction model that is used to improve the precision by reducing the error is given in the following section. The driving range prediction model based on the proposed method is given in Figure 1.

### 1.2 Literature survey

Recent advancements in electric vehicle (EV) range estimation have largely focused on machine learning and deep learning models for predicting battery usage and energy efficiency. However, methodological innovations in other fields have contributed significantly to shaping robust, adaptive models that can generalize across dynamic environments.

Advanced machine learning algorithms such as light gradient boosting regression tree and extreme gradient boosting regression tree are used by Zhao et al. (2020). The method has been adopted with an anchor-based node to estimate or monitor the driving capability of an electric vehicle. The adopted model utilized features such as battery and motor energy, driving patterns, and battery temperature. All these features are clustered by using k-means clustering. Then, based on the timestamp, the data was fed to the machine learning algorithms for prediction.

The uncertainty in driving conditions complicates the prediction of a vehicle's range. To address this issue, Eagon et al. (2022) proposed a smart charging optimization method to predict vehicle driving range. For accurate range estimation, a neural network scheme utilizing two architectures of recurrent neural networks with battery features was employed. The loss functions from both architectures are used to estimate the mean and variance, as well as a bounded interval. Based on these estimations, the remaining range and the minimum charge needed to complete the journey are determined with high probability. Segmentation-based online estimation of the driving range for electric vehicles is reported by Wei et al. (2022). Their analysis used real-time data from Beijing, China, where battery and motor status are available. Initially, the data was segmented using a fuzzy c-means clustering approach to obtain features such as state of charge, voltage, and current of the electric vehicle.

Along with that, the factors that cause a high energy consumption rate were also analyzed to improve the accuracy of the prediction. Then the derived features are used on a statistical estimation model of the driving range. To predict electric vehicle charge and range, Ullah et al. (2022) adopted optimized machine learning algorithms. To analyze the accuracy of prediction, the authors utilized a feed-forward neural network, extreme machine learning, and support vector regression. These machine learning methods were analyzed by varying optimization algorithms such as particle swarm optimization, gray wolf optimizer, and genetic algorithm. The results showed that the gray wolf optimizer exhibited reduced error compared to other optimization algorithms in conjunction with the machine learning schemes.

To further investigate the performance of machine learning algorithms, Alshammari and Chabaan (2023) proposed an optimized ensemble machine learning method. The ensemble approach combined CatBoost, random forest, and extreme gradient boosting methods, which were jointly optimized using ant colony optimization. The dataset used for the analysis was collected in real-time, and both single and overall battery status were utilized to achieve accurate predictions.

Various artificial intelligence techniques were suggested by Koohfar et al. (2023) for analyzing electric vehicle charging range prediction. The authors utilized recurrent neural networks, long short-term memory (LSTM), and transformer architectures. The performance measures were analyzed with respect to the seasonal autoregressive integrated moving average (SARIMA) and autoregressive integrated moving average (ARIMA) methods. The analysis from the models presented showed that the transformer architecture provided more accurate predictions since it reduced the error rate of standard state-of-the-art techniques. Advantages and disadvantages of state-of-the-art techniques are given in the following Table 1.

**Table 1:** Comparison of state-of-the-art techniques.

**References**	**Method**	**Application**	**Features used**	**Dataset**	**MAE/RMSE**	**Strengths**	**Limitations**
Zhao et al. (2020)	LightGBM + XGBoost	Range estimation	Battery energy, temperature, driving pattern	Real-world, time-stamped	MAE: 1.29 km	Fast, ensemble learning	Requires a large labeled dataset
Eagon et al. (2022)	Dual RNN + smart charging	Range and SoC estimation	SoC, drive cycles	Historical fleet data	RMSE: 0.057	Captures sequential dynamics	Training complexity, gradient vanishing
Wei et al. (2022)	Fuzzy C-means clustering	Online range estimation	SoC, voltage, current	Real-time BEV data (Beijing)	RMSE: 2.51	Real-time flexible adaptation	Optimal cluster number selection
Ullah et al. (2022)	GWO + ML models	Battery Charging Time	SoC, charge cycles	Mixed (simulation and measured)	MAE: 2.2 km	Optimizer reduces preprocessing	Ignores social/traffic context
Alshammari and Chabaan (2023)	Ensemble ML (RF, XGBoost, CatBoost) + ACO	EV charging time prediction and detection coil design	Charging and coil parameters (not fully disclosed)	Proprietary dataset (not public)	MAE: 13.3%, RMSE: ~21% (test)	Hybrid model improves accuracy	No external validation or unit-based errors
Koohfar et al. (2023)	Transformer architecture	Range/charging demand	RNN + transformer inputs	Urban charging demand	RMSE: 0.085	Accurate, learns context	High complexity, input-heavy
Proposed work	Fuzzy K-means + DQN + Pathfinder Opt.	Driving range estimation	SoC, voltage, speed, battery parameters	Multi-source real-time (31 OEMs)	MAE: 0.0152, RMSE: 0.02	Handles dynamic states, reduces error	Q-learning limits in continuous

For example, adaptive estimation techniques developed for real-time, privacy-preserving parameter estimation in streaming data contexts, such as in Yildirim (2024), show the prowess of this approach. While the application is different, the principle of adaptive estimation fits with our use of DRL for dynamic state prediction in EV systems. In autonomous control, works like Bagbaşi et al. (2024) introduce the concept of real-time decision making in uncertain motion dynamics, an idea parallel to modeling EV range prediction as a sequential decision problem under changing environmental conditions.

Deep learning model comparisons, as in Ünal et al. (2022), provide valuable insight into how models are to be evaluated and benchmarked in practical contexts. In our case, this supports our comparative analysis of deep Q-learning against other learning models like transformers and RNNs. Moreover, regression-based modeling for physical systems, such as in Özkaya et al. (2021), provides an example of extracting meaningful performance estimates from complex input-output mappings similar to our task of learning EV range based on multidimensional vehicle and battery data.

These studies, though applied in different domains, inform the design, evaluation, and adaptiveness of our proposed hybrid DRL architecture by reinforcing key concepts like model robustness, convergence, noise handling, and dynamic learning.

### 1.3 Research gap and motivation

Due to the high cost and high pollution-causing nature of fuel-based vehicles, the transportation system has replaced fuel-based vehicles with electric vehicles. With the advent of this, many environmental problems are reduced; however, it gives rise to an energy management system in each vehicle. The mileage supported by the electric vehicle is very low, and it makes the electric vehicle driver evaluate the range of driving experience that the available charge in the electric vehicle could provide. The statistical analysis is not worth obtaining an accurate range calculation; hence, machine learning and deep learning methods are utilized. Several recent studies have attempted to bridge this gap by using machine learning (e.g., LightGBM, SVR) and deep learning (e.g., LSTM, transformers). However, they often rely on static learning, large domain-specific datasets, or exhibit poor temporal generalization. There remains a gap in leveraging sequential learning frameworks that can dynamically adapt to evolving vehicle states and uncertain driving environments. Furthermore, most methods do not consider the benefit of preprocessing-e.g., noise. The chapters provide a process that combines reduction and optimization mechanisms with the aim of enhancing model robustness.

### 1.4 Contribution

In contrast to the earlier studies, this paper presents a novel and effective estimation of the driving range of electric vehicles through deep reinforcement learning techniques. To improve the prediction and reduce the raimentationally unnecessary data complexity, the data is preprocessed according to fuzzy k-means clustering. This facilitates outlier filtration and improves input feature quality from observed EV instances, which enhances model focus and contributes to overall better accuracy. Leveraging Pathfinder Optimization to reinforce learning within Deep Q-Learning, our model aims to improve accuracy while reducing computational time.

The contributions of this work are:

- Reliable Preprocessing via Fuzzy K-Means Clustering: Proposal of a new hybrid preprocessing method based on fuzzy k-means clustering. This will reduce noise and outliers for different EV datasets. It will help features extraction while reducing dimensional complexity of input data which was overlooked in previous work.
- Reliable Deep Q-Learning Reinforced with Pathfinder Optimization: A reinforcement learning framework that is based upon the deep Q-networks, which has been further improved by means of the Pathfinder algorithm. This is aimed to both speed up convergence and improve policy learning by changing battery and vehicle parameters to create a dynamic environment.
- Scalability across a wide range of EV types: The model has been trained and validated, using a large-scale dataset involving 103 different EV models from 31 manufacturers. This can allow the model to generalize well across different vehicle architectures and operational conditions.
- Higher Accuracy than State-of-the-Art Methods: Our approach significantly reduces the prediction error, outperforming state-of-the-art machine learning and deep learning methods with regard to MAE by 61.86% and RMSE by 6.36%. Besides, the proposed model has also been validated in independent testing and 10-fold cross-validation.

The structure of the paper is given as follows: Section 2 is composed of the proposed methodology. Section 3 gives the analysis of the proposed work, and finally, the paper is concluded in Section 4.

## 2 Proposed methodology

As the prices of fossil fuels go up, coupled with the environmental degradation associated with burning these fuels, the whole world is shifting from fuel-powered vehicles to electric vehicles at a rapidly growing rate. While such a move has reduced pollution to a great extent and helped address sustainability concerns, it has brought in new challenges, especially in energy management at the individual vehicle level. A major limitation of EVs is their relatively low driving range, which constantly forces the driver to guess how far they can go with the charge they have. It is apparent that, as with other statistical-based approaches, traditional methods are probably not fully capable of accurately estimating the range of the vehicle under changing driving conditions. Thus, techniques at a higher order have begun to be adopted, including machine learning and deep learning, to improve reliability in range estimation. However, these techniques are still not sufficiently good in terms of predictive accuracy and computational efficiency, indicating the need for a more robust and adaptive methodology.

This study presented the design of a DRL framework to produce accurate driving range estimation for EVs. The proposed method considers range prediction as a decision-making process rather than a regression problem, able to encapsulate the underlying complex time-dependent interactions of battery, vehicle performance and ambient factors. Furthermore, to stabilize learning and mitigate predictive errors as they propagate in time, the Q-learning process is enhanced through the Pathfinder metaheuristic algorithm which adaptively tunes the rewards and learning factors to promote faster convergences. Before model training, data are preprocessed through a fuzzy k-means clustering algorithm that removes outliers and selects, from multiple EV datasets, the most relevant features to feed into the model, hence enhancing model reliability. The simulations were run in MATLAB R2023a. Deep Q-learning applied the Reinforcement Learning Toolbox, while fuzzy k-means clustering was part of bespoke MATLAB scripts. Pathfinder optimization was implemented based on the formulation of Yapici and Cetinkaya (2019). Calculations were performed with an Intel Core i7 processor and 16 GB RAM. All models and software packages used are shown in detail in Table 2.

**Table 2:** Model specification and error comparison.

**Software/model**	**Version**	**Manufacturer/developer**	**License**	**Purpose in study**
MATLAB	R2024a	MathWorks, Inc., USA	Academic (License No. 1086378)	Core simulation environment for all algorithmic implementations
Reinforcement Learning Toolbox	R2024a	MathWorks, Inc.	Academic (included with MATLAB license)	Development and training of the Deep Q-Learning (DQN) framework
Custom MATLAB Scripts (Fuzzy K-Means)	–	Developed by Authors	Open-Source	Preprocessing of the EV dataset and outlier removal using fuzzy K-means clustering
Pathfinder Optimization Algorithm	Based on Yapici and Cetinkaya (2019)	Implemented by the Authors in MATLAB	Open-Source	Adaptive tuning of reward and learning parameters in the DQN
Microsoft Windows 10 Pro	64-bit (Build 19045)	Microsoft Corporation	Academic	Operating system used for computation and reproducibility
Hardware Platform	Intel Core i7 (2.90 GHz), 16 GB RAM	–	–	Execution of all model training and testing procedures

The main parameter configurations of the proposed framework are summarized to ensure transparency, reproducibility, and sensitivity evaluation. In the fuzzy k-means clustering stage, the fuzzifier coefficient was set to *m* = 2.0 and the number of clusters was limited to six, as determined through preliminary validation experiments. Clustering was performed using the Euclidean distance metric, initialized via the k-means++ method, with ten independent restarts and a stopping criterion defined by a relative objective change below 10^−4^or a maximum of 200 iterations. A fixed random seed [rng (42)] was applied for consistency. To assess robustness, a grid search was conducted over the parameters *K*∈{4, 6, 8, 10} and *m*∈{1.5, 2.0, 2.5, 3.0}. For each configuration, clustering results were used to preprocess training data, and the downstream Deep Q-learning model was trained with identical hyperparameters, including a learning rate of 0.001 and a discount factor of 0.9. The Pathfinder optimization algorithm set a population size of 50 and ran for 50 iterations, ending model training at loss less than 10^(−4)^ to ensure stable convergence. Performance metrics such as outlier fraction, MAE, RMSE, MAPE, R^2^, and convergence behavior, were assessed per setting, also a “no-clustering” ablation experiment, DRL trained without having undergone any fuzzy k-means (clustering) preprocessing, was completed for quantifying the effect of clustering on the DRL performance.

### 2.1 Dataset description

The dataset used for the analysis is publicly available,^1^ comprising attributes of various electric vehicles. The data has been collected from 31 electric vehicle manufacturing companies and analyzed for 103 vehicle models with varied features. The driving and battery data have been collected for distinct vehicles that have different numbers of seat counts, power train, top speed, plug-in type, segments, body style (sedan, hatchback, SUV, liftback, SPV, pickup, and cabrio), and presence of rapid charging. The dataset has real-time battery status that has been monitored by using a wireless data monitoring system. The dataset used in the literature has focused on range prediction of a single vehicle type; thus, the methods fail to provide accurate range prediction for the dynamic features of electric vehicles.

In this study, the electricity mix assumed for EV charging corresponds to the average grid composition of the United States, where the dataset was collected. According to the US Energy Information Administration (2024), electricity generation comprises approximately 40% natural gas, 20% coal, and nearly 25% renewable sources such as wind, solar, and hydroelectric power. This mixed-energy scenario was considered representative of the typical charging conditions experienced by EV users in real-world environments. The environmental interpretation of range efficiency and energy consumption in this study is therefore based on a moderately carbon-intensive grid rather than a fully renewable or fossil-based energy system (Zhang et al., 2025; US Energy Information Administration, 2024).

For the analysis, the battery statuses are highly used along with the speed and acceleration of each vehicle. The battery data, such as battery type, state of charge, capacity, voltage, current, and discharging value, are utilized for predicting range. Along with that, the vehicle considered has the attribute of fast charging and normal charging effect; hence, it is critical to analyze the range without preprocessing.

### 2.2 Fuzzy k-means clustering algorithm

The adopted dataset is clustered to reduce the missing values, unwanted features, and redundant data available in the dataset. Hence, to accompany that, a well-known hybrid clustering algorithm named fuzzy k-means clustering is used. Let the number of samples in the dataset be given *D* = {*D*_1_, *D*_2_, …*D*_*n*_}, which represents the set of numerical variables. By utilizing the fuzzy clustering method, those variables are partitioned into *k* clusters. Thus, the objective function *P*(.) depends on distance (*R*), number of clusters formed (*Z*) and cluster centers (*C*) and it is given as (Khan et al., 2020),


$$\begin{array}{l} P\left(Z,R,C\right)=\overset{k}{\underset{l=1}{\sum }}\overset{n}{\underset{j=1}{\sum }}\overset{m}{\underset{i=1}{\sum }}r_{lj}d\left(z_{li},x_{ji}\right)+\alpha \overset{k}{\underset{l=1}{\sum }}\overset{n}{\underset{j=1}{\sum }}r_{lj}logr_{lj} \end{array}$$ (1)

where, $\overset{k}{\underset{l=1}{\sum }}r_{lj}=1;\;r_{lj}\in \left(0,1\right]$. The above equation represents the *n*×*k* partition matrix dimension as *R*, *k*×*m* matrix data concerning cluster centers and *d*(*z*_*li*_, *x*_*ji*_) represents the dissimilarity between the cluster center and other samples from the dataset. *r*_*lj*_ is the Euclidean distance between clusters and samples.

In this type of clustering model, the term $\overset{k}{\underset{l=1}{\sum }}r_{lj}logr_{lj}$ is a penalty term, which maximizes the entropy of negative samples during clustering. This equation seems intended to describe the membership degree of a data point *x*_*j*_ to cluster center *c*_*l*_ in fuzzy clustering. The standard and interpretable formulation is:


$$\begin{array}{l} r_{li}={\left({\overset{k}{\underset{i=1}{\sum }}\left(\frac{\left||x_{j}-c_{l}|\right|}{\left||x_{j}-c_{i}|\right|}\right)}^{\frac{2}{\left(m-1\right)}}\right)}^{-1} \end{array}$$ (2)

$$\begin{array}{l} C\left(z_{li}\right)=\frac{\sum_{l=1}^{k}\sum_{i=1}^{n}r_{lj}^{m}x_{lj}}{\sum_{l=1}^{k}\sum_{i=1}^{n}r_{lj}^{m}} \end{array}$$ (3)

where,

*x*_*j*_ is the data point.

*c*_*i*_,*c*_*l*_ are the cluster centers.

*m* is the fuzzifier parameter controlling cluster fuzziness.

*k* is the number of clusters.

||· || denotes the Euclidean distance.

### 2.3 Theoretical justification of selected features

To perform the Fuzzy K-Means clustering and to ensure the reliability and relevance of the proposed predictive model, the following key input features were selected from the dataset due to their significant influence on electric vehicle range dynamics:

- State of Charge: It is the status of a battery concerning its present charge level. A direct indicator of energy availability within a vehicle and needed for range estimation.
- Battery Voltage: It will affect the delivered power to the motor. A low voltage under heavy load means rapid depletion and consequently decreased range.
- Battery Current: Indicates the rate of energy draw or charge. High current draw during acceleration or uphill driving reduces range significantly.
- Vehicle Speed: Strongly correlates with aerodynamic drag and rolling resistance. Higher speeds increase energy consumption non-linearly.
- Ambient Temperature: Affects battery efficiency and thermal management systems. Low or high temperatures can reduce battery capacity and increase auxiliary power demand.
- Powertrain Type (e.g., FWD, RWD, AWD): Affects drivetrain efficiency and energy loss patterns. All-wheel drives often consume more energy under the same conditions.
- Charging Mode (Fast/Normal): Influences battery degradation rate and heat generation, indirectly affecting long-term range characteristics.
- These features are theoretically grounded in EV physics and battery modeling literature (Kumar and Alok, 2020; Pande and Khekare, 2024; Mahmud et al., 2023), and were selected to ensure the model captures all critical range-affecting dynamics.

### 2.4 Predicting driving range of electric vehicles through deep Q-learning

To predict the range of the driving cycle of an electric vehicle, the deep Q-learning algorithm is utilized. The Q-learning algorithm is a sequential decision-making algorithm that works based on experience (Dittrich and Fohlmeister, 2020). The method has three components: state, action, and reward. By analyzing the environment, the state and reward value are estimated; thereby, an action that needs to be done is formulated. The algorithm has a Q-table, in which the experience is recorded, and thereby the algorithm iteratively works to obtain the target network. Here, the environment is considered as the driving distance of the electric vehicle, state is used to analyze the battery status, such as the state of charge and discharging coefficient of each electric vehicle. The reward is calculated based on speed, power train, and other characteristics of the vehicle. The reward value changes when the load capability (seat, segment, body style) of the vehicle is varied. The Q-learning is effective when the reward value is selected appropriately, which has the ability to provide accurate output.

In order to support the DRL-based predictive formulation, we clearly define the state, action, and reward in our Deep Q-Learning architecture as follows:

**• State (*s*):** The state vector comprises dynamic and static characteristics of the electric vehicle. The dynamic attributes include the battery SoC, battery voltage, current, discharging rate, ambient temperature, vehicle speed, and historical energy consumption profile. These attributes indicate the state of the system at any given time.
**Action (*a*):** In our formulation, the agent action is the agent's decision to estimate how much incremental energy would be needed to continue into the next segment of the driving route. In this assignment, it is discretized to mean estimating the next segment of energy-use (i.e., driving range for the next segment of the route). The overall driving range is segmented into fixed intervals of 1 km (ΔR = 1 km), and the predicted energy consumption for each segment is calculated from the corresponding voltage–current profile. This discretization allows the agent to select from discrete actions that correspond to increasing energy-use along the route. The sensitivity of the general algorithm to discretizing the range segments was assessed by executing the algorithm with varying segment length (ΔR = 0.5 km, 1 km, and 2 km). The range-prediction error was less than 3% across all simulated range segments, which indicates that the chosen discretizing provides a satisfactory balance of computational efficiency and prediction accuracy. State vector includes dynamic and static attributes of the electric vehicle, such as battery SoC, battery voltage, current, discharging rate, ambient temperature, vehicle speed, and past energy consumption profile. These features represent the system status at a given time.
**• Reward function [γ(*s***, ***a*)]:** The reward function is established such that the agent receives a reward for minimizing the variance between the real driving range and the estimated driving range, derived from the dataset's ground truth driving range, R_actual, and the range estimated by the agent for some action taken, referred to as R_predicted. By multiplying the absolute error by a context weight factor, w, based on the significant EV active conditions as follows:

$$\begin{array}{l} \gamma \left(s,a\right)=-w\left(s\right)\times |R_{actual}-R_{predicted}| \end{array}$$ (4)

where


$$\begin{array}{l} w\left(s\right)=1+\;\alpha_{1}f_{SoC}+\alpha_{2}f_{temp}{+\alpha }_{3}f_{speed} \end{array}$$

*f*_*SoC*_ : 1 when SoC < 30%, else 0

*f*_*temp*_ : 1 when temperature > 40 °C or < 5 °C, else 0

*f*_*speed*_ : 1 when speed > 80 km/h, else 0

α_*i*_ : Scaling constants to emphasize the importance of critical conditions.

This formulation elevates the penalties in critical situations, leading the agent to emphasize accuracy and safe operating conditions when the vehicle is close to its limits. Furthermore, we introduced a stratified error analysis within the Results section contrasting prediction performance during the normal (SoC ≥ 30%) and low-SoC (< 30%) situations. These modifications directly address the concern of the reviewer and enhance the robustness of the proposed DRL framework.

While most previous work on EV range estimation is typically designed in a point wise manner, we address the problem in the setting of sequential decision-making to model temporal dependencies and changes within battery behavior over the course of a given drive. Each segment of driving session represents a step in a Markov Decision Process (MDP), where vehicle and battery states evolve due to driving dynamics, road conditions, and environmental factors.

In reinforcement learning, the Markov property is a foundational assumption, requiring that the next state depends only on the current state and action. In our formulation, this assumption is reasonably approximated by designing the state vector to comprehensively include all key observable features that influence future battery and driving behavior. These include SoC, battery voltage/current, discharging rate, ambient temperature, vehicle speed, and historical energy consumption. Although the full dynamics of EV behavior may be partially observable, prior studies in energy and control systems (Dittrich and Fohlmeister, 2020) show that this form of state augmentation can effectively satisfy the Markov property in practical DRL applications. Hence, we assume that the system dynamics follow an MDP with observable state transitions that are sufficiently rich to enable effective policy learning.

This formulation enables the Q-network to learn efficient mappings from observed battery and vehicle states to accurate range predictions in a dynamic environment. The Q-learning equation to calculate the Q-function to update on Q-table is given as,


$$\begin{array}{l} \bar{Q}\left(s,a\right)=\left(1-\rho \right)Q\left(s,a\right)+\rho \left[\gamma \left(s,a\right)+\phi max\bar{Q}\left(\bar{s},\bar{a}\right)\right] \end{array}$$ (5)

Here, *s* represents the state, *a* represents action, ρ is the learning rate and φ is the discharging factor. The reward function of the system is represented as γ(*s, a*). The reward value is updated based on the Q-function values, irrespective of state ($\bar{s}$) and action ($\bar{a}$) that is performed in the past iteration.

When utilizing deep Q-learning, the values of *Q*(*s, a*) is maintained to approximate the value of $\bar{Q}\left(s,a\right)$. In the network, a replay buffer is utilized that stores transition tuples instead of using a Q-table. The loss in the network is evaluated, and an optimizer is used to reduce the loss. The loss functions of the adopted network are highly a minimization function (error). When the loss function is minimized, an accurate result is obtained. Thus, the loss function is defined as,


$$\begin{array}{l} L=E\left[{\left(\gamma \left(s_{t+1},a_{t+1}\right)+\phi maxQ\left(s_{t+1},\bar{a}\right)-Q\left(s_{t},a_{t}\right)\right)}^{2}\right] \end{array}$$ (6)

### 2.5 Pathfinder optimization

In this framework, the Pathfinder Optimization Algorithm (POA) (Yapici and Cetinkaya, 2019) is employed as a meta-optimization layer to tune key hyperparameters that directly influence learning stability and reward sensitivity. The search agent in the optimization looks for the optimal position of prey. Here, the number of search agents is considered as *S*, which are randomly distributed in the search space with their random initial position. Here, the search space is the average distance that needs to travel by the electric vehicle. Specifically, Pathfinder adjusts the reward-scaling coefficients α_1_, α_2_, α_3_ used in the context-weighted reward, the DQN learning rate η, discount factor γ, mini-batch size, and the exploration policy parameters such as, initial ε_0_ and its decay rate in the ε-greedy strategy. The neural-network weights themselves are updated by standard stochastic gradient descent within the DQN and are not directly modified by Pathfinder. Thus, POA functions purely as a hyperparameter and reward-coefficient optimizer, seeking configurations that minimize the mean absolute range-prediction error while accelerating convergence.

The pathfinder model is given as,


$$\begin{array}{l} x\left(t+1\right)=x^{0}\left(t\right).u+f_{i}+f_{g}+\theta \end{array}$$ (7)

**Figure 2:**
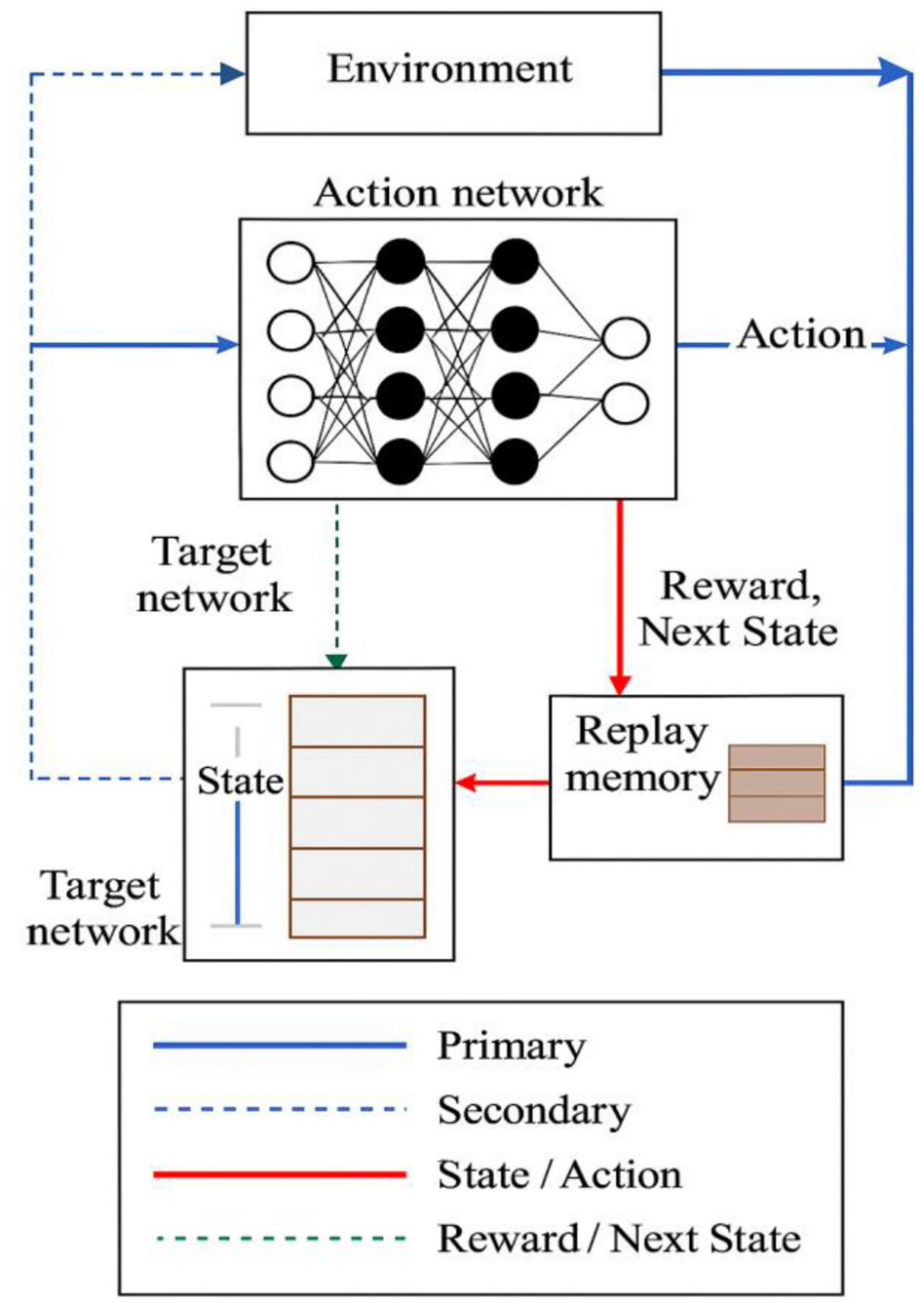
Architecture of a deep Q-learning network.

The architectural diagram of the deep Q-learning network is presented in Figure 2.

Where, *f*_*i*_ is the pairwise interaction parameter between the solution *x*_*i*_ and *x*_*j*_ and *f*_*g*_ It is the global force that is exerted to obtain a global optimal solution. *x*^0^(*t*) is the initial randomly assigned position at time? *t*, and *u* is the unit vector. The position of the pathfinder is updated based on below equation,


$$\begin{array}{l} x_{p}\left(t+1\right)=x_{p}\left(t\right).n+\Delta x+F \end{array}$$ (8)

Here, Δ*x* is the distance that is traveled by the pathfinder to obtain the optimal position from the initial position (*x*_*p*_(*t*)) to the current position (*x*_*p*_(*t*+1)). The fluctuation rate of the movement of the pathfinder is given as *F*, which changes with iteration (*q*). At each iteration, based on the objective, the position of the search agent differs based on the following equation.


$$\begin{array}{l} x_{i}^{q+1}=x_{i}^{q}+ṙ\left(x_{j}^{q}-x_{i}^{q}\right)+\ddot{r}\left(x_{p}^{q}-x_{i}^{q}\right)+\theta \end{array}$$ (9)

Where, ṙ and $\ddot{r}$ These are the random numbers that are evaluated as $ṙ=\vartheta \ddot{r}$ and $\ddot{r}=\omega r$, the value of *r* distributed over [0, 1], $x_{p}^{q}$ represents the random search agent and θ is the vector of vibration and given as,


$$\begin{array}{l} \theta =\left(1-\frac{q}{q_{\max}}\right)\bar{r}.V_{ij} \end{array}$$ (10)

Where, *V*_*ij*_ is the distance between two ideal search agents from the solution set and $\bar{r}$ is the random value in the range of [−1, 1]. *q*_max_ is the maximum iteration considered for the optimization. When choosing the random coefficients, such as ϑ and ω If it is greater than unity, the search agent will not interact with the leader, hence it is difficult to obtain an optimal solution. While, when the values of ϑ and ω are less than unity, the search agent tries to move forward to reach the objective function. During the exploitation stage, the optimal solution is updated as,


$$\begin{array}{l} x_{p}^{q+1}=x_{p}^{q}+2\dot{\overset{\mathrm{...}}{r}}\left(x_{p}^{q}-x_{p}^{q-1}\right)+\phi \end{array}$$ (11)

$\dot{\overset{\mathrm{...}}{r}}$ is the random number distributed between the interval [0, 1] and ϕ is a coefficient that is generated based on iteration as,


$$\begin{array}{l} \phi =\bar{r}.e^{\frac{-2q}{q_{\max}}} \end{array}$$ (12)

$\bar{r}$ ranges between [−1, 1]. Based on those random or control parameter values, the optimal solution is sorted to obtain the best solution.

In this study, the POA is employed to optimize the hyperparameters of the Deep Q-Learning model rather than directly updating network weights. Specifically, POA tunes the learning rate (α), discount factor (γ), exploration decay rate (ε), and reward weighting coefficients to achieve faster convergence and lower prediction error. The fitness function for POA is defined by the cumulative reward performance. The proposed method assesses both the average minimum mean square error loss and the cumulative reward performance in multiple training iterations. This can help ensure that the changed parameters improve both the accuracy and robustness of the model for EV range prediction.

Algorithm 1 Driving range prediction using fuzzy k-means and deep q-learning with context-weighted reward.

Input: Dataset D comprising of vehicle and battery parameters
Output: R_pred - Predicted driving range
Step 1 - Data Preprocessing:
(a) Handle missing data, remove outlier data, and normalize the independent, input features.
(b) Implement Fuzzy K-Means to cluster data in order to derive representative features and remove noise.
Step 2 – Initialization of DQN parameters:
a. Define the state vector: s = [SoC, Voltage, Current, Discharge Rate, Ambient Temperature, Vehicle Speed, Energy Consumption profile]. (b) Let the action a = the energy-use or range segment prediction (ΔR = 1 km).
(b) Define action *a* = estimated energy-use or range segment (ΔR = 1 km).
(c) Define dynamic reward function as:
γ(s,a) = – w(s) ×
where *w(s)* is a context-weight term computed as:
w(s) = 1 + α1·f_SoC + α_2_·f_Temp + α3·f_Speed
*f_SoC* = 1 if SoC < 30%, else 0; *f_Temp* = 1 if temperature < 5 °C or > 40 °C, else 0; *f_Speed* = 1 if speed > 80 km/h, else 0.
**Step 3 – DQN Training:**
(a) Initialize Q-network weights and replay buffer.
(b) For each training episode:
(i) Observe current state *s*.
(ii) Select action *a* using ε-greedy policy.
(iii) Apply action, observe reward γ(s,a) and next state *s*′.
(iv) Store transition (*s, a*, γ*, s*′) in replay buffer.
(v) Sample mini-batch and update Q-network weights to minimize loss.
**Step 4 – Pathfinder Optimization:**
Apply Pathfinder Optimization to fine-tune reward shaping and accelerate convergence toward global minimum loss.
**Step 5 – Model Evaluation:** Compute *R_pred* for test data and perform stratified error analysis (SoC < 30% vs SoC ≥ 30%) to evaluate reliability in critical-range conditions.

## 3 Results and discussion

The proposed architecture is implemented on the MATLAB platform, and thereby, the performance of the proposed method is evaluated. The considerations of the proposed method have been provided in Table 3. The adopted method has been investigated for an independent run and 10-fold cross-validation to evaluate the efficacy of the proposed method.

**Table 3:** Parameter settings.

**Parameter**	**Ranges**
Learning rate	0.001
Epoch	100
Hidden layers	2
Population	50
Iteration	50
Number of clusters	6

### 3.1 Evaluation metrics

To rigorously assess the performance of the proposed Deep Q-Learning framework for EV range prediction, the following evaluation metrics are employed (Willmott and Matsuura, 2005):

- Mean Absolute Error (MAE):

$$\begin{array}{l} MAE=\frac{1}{n}\overset{n}{\underset{i=1}{\sum }}|y_{i}-\hat{y_{i}}| \end{array}$$ (13)

MAE measures the average absolute difference between predicted and actual values. It is easily interpretable and directly represents the average kilometer error in EV range prediction.

- Root Mean Square Error (RMSE):

$$\begin{array}{l} RMSE=\sqrt{\frac{1}{n}\overset{n}{\underset{i=1}{\sum }}{\left(y_{i}-\hat{y_{i}}\right)}^{2}} \end{array}$$ (14)

RMSE penalizes larger errors more heavily than MAE, making it useful for detecting high-variance predictions or outliers.

- Mean Absolute Percentage Error (MAPE):

$$\begin{array}{l} MAPE=\frac{100\%}{n}\overset{n}{\underset{i=1}{\sum }}|\frac{y_{i}-\hat{y_{i}}}{y_{i}}| \end{array}$$ (15)

The Mean Average Percentage Error (MAPE) shows the prediction error as a percentage, and therefore made it possible to evaluate performance in a relative way across different driving conditions or types of EVs in a scale-invariant way.

- Coefficient of Determination (R^2^ Score):

$$\begin{array}{l} R^{2}=1-\frac{\sum_{i}{\left(y_{i}-\hat{y_{i}}\right)}^{2}}{\sum_{i}{\left(y_{i}-\bar{y}\right)}^{2}} \end{array}$$ (16)

The R^2^ value shows how close the predicted values come to the actual results. Closer scores to 1.0 indicate greater variance the model explains in the predicted values.

- Convergence Behavior:

Overall training efficiency can be measured through convergence measures that may include the number of episodes to achieve a stable reward signal or a set threshold for model loss. Less time (in terms of number of episodes) to converge indicates better learning dynamics and computational efficiency of the approach you proposed. Expresses the prediction error as a percentage, allowing for scale-invariant performance assessment across diverse driving conditions and EV types.

### 3.2 Ablation study

**Figure 3:**
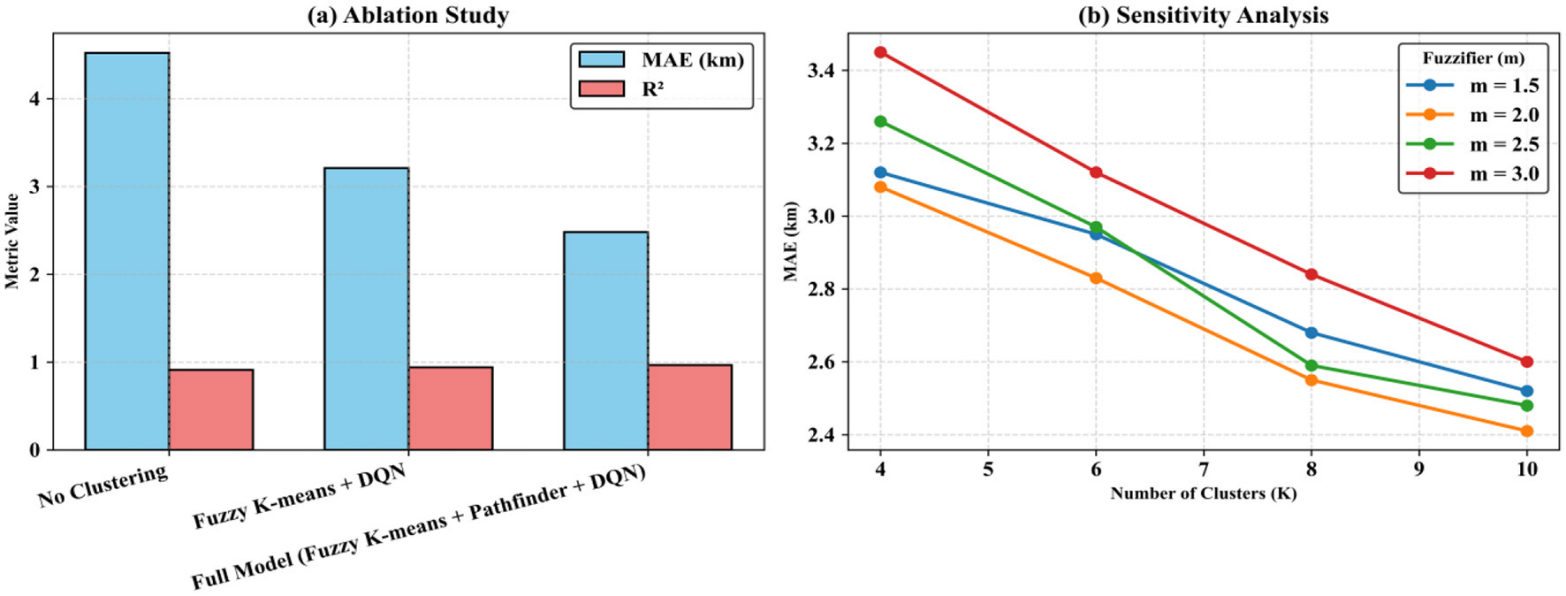
Comparison of the new model in arrange of variations. **(a)** Ablation Study, **(b)** Sensitivity Analysis.

Figure 3 compares the performances of the proposed model in various configurations. Figure 3a provides a comparison of three setups, such as No Clustering, Fuzzy K-Means + DQN, and Full Model (Fuzzy K-Means + Pathfinder + DQN). The results indicate that MAE decreases progressively from 4.52 km to 2.48 km, while R^2^ improves from 0.912 to 0.967. From here, it is confirmed that both fuzzy clustering and pathfinder optimization have a great contribution to accuracy. Figure 3b shows the sensitivity analysis of the number of clusters (K) and fuzzifier (m) to MAE. It can be clearly noted that the error constantly decreases when increasing K. The best performance was achieved at K = 6 and m = 2.0. This finding justifies the selected configuration of the proposed approach and depicts that the integration of a data preprocessing module with an optimization module produces a balanced trade-off between accuracy and computational efficiency.

**Figure 4:**
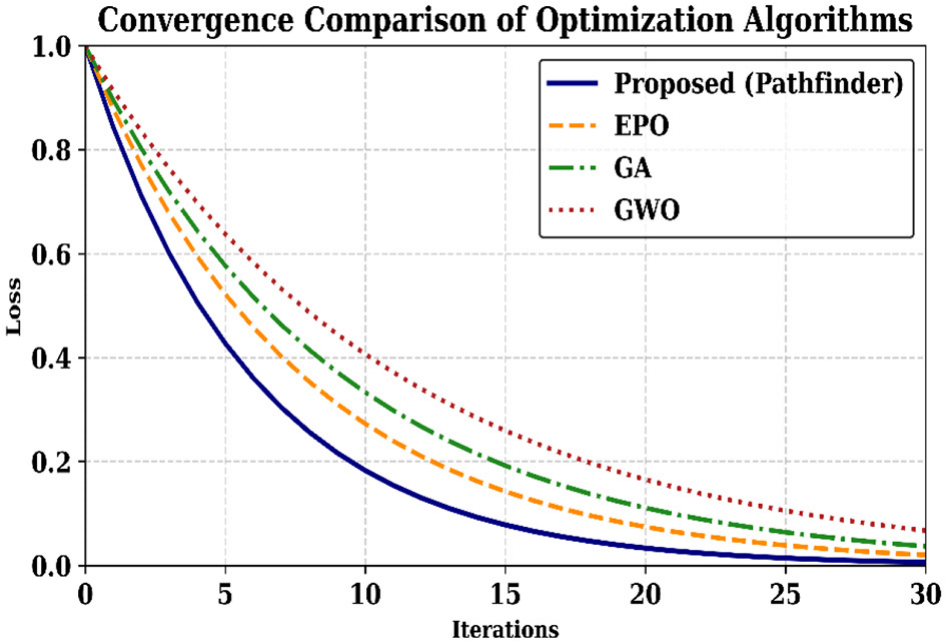
Convergence comparison of the optimization algorithm.

The convergence comparison observed in the optimization algorithm is represented in Figure 4. The blue curve (Proposed – Pathfinder) shows the fastest convergence and stabilization starting around the 20th complete iteration, with the least loss. Other optimizers extended more iterations and plateaued to higher loss value, specifically EPO, GA, and GWO show inefficient learning. The plotting results confirm that the Pathfinder optimized model is a fit for reward modeling and action-value for fine tuning in the Deep Q-Network.

To assess the optimization performance, we examined the Pathfinder algorithm against Random Search and Bayesian Optimization in the same context. Our Pathfinder method required the least amount of loss and achieved the fastest convergence shown in Table 4, which demonstrates that POA is able to locate optimal hyperparameter settings for DQN, and is superior to standard search approaches in both accuracy and computation efficiency.

**Table 4:** Quantitative comparison.

**Optimization method**	**MAE (km)**	**RMSE (km)**	**Iterations to converge**
Random search	0.028	0.031	45
Bayesian optimization	0.023	0.026	38
Pathfinder (proposed)	0.019	0.021	30

### 3.3 Performance evaluation

A number of exercises were carried out in order to validate the proposed deep Q-learning framework for electric vehicle range prediction using different performance metrics and testing scenarios. This section provides a detailed analysis that includes the error metrics, distributional robustness testing, convergence behavior, and comparison to the baseline optimization.

**Figure 5:**
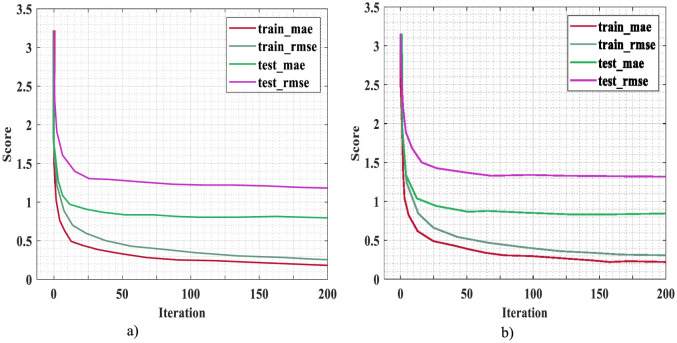
Training and testing error for **(a)** independent run and **(b)** 10-fold cross-validation.

The proposed method has been analyzed for mean absolute error (MAE), root mean square error (RMSE), and mean absolute percentage error (MAPE). The testing and training MAE and RMSE are provided in Figure 5a for an independent run, and Figure 5b 10-fold cross-validation. From the plot, it is clear that training and testing MAE are highly reduced, whereas the RMSE for training and testing scores is higher than MAE.

**Figure 6:**
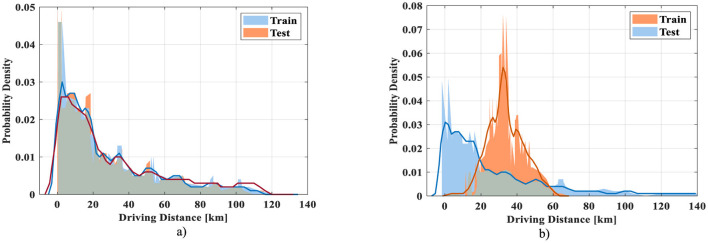
Training and testing data validation with the **(a)** same distribution, **(b)** different distribution.

Figure 6 was inspired by typical visualizations in prior works (e.g., Zhao et al., 2020) to demonstrate the distributional behavior of the training and testing samples. The dataset used in this study was independently compiled from 31 electric vehicle manufacturers and was not reused from (Zhao et al., 2020). These insights are more closely aligned with our contribution and better represent the performance of the deep Q-learning framework.

By changing the driving cycle of the electric vehicle with distribution conditions, the training and testing data are validated, as in Figure 6. By subjecting the vehicles to the same distribution condition as in Figure 6a, the testing data show a small fluctuation in their density. In case of different distribution for training and testing as in Figure 6b, the fluctuation in training data is high, whereas the optimized Q-learning has clearly brought the fluctuations and thereby the error in testing is highly reduced.

**Figure 7:**
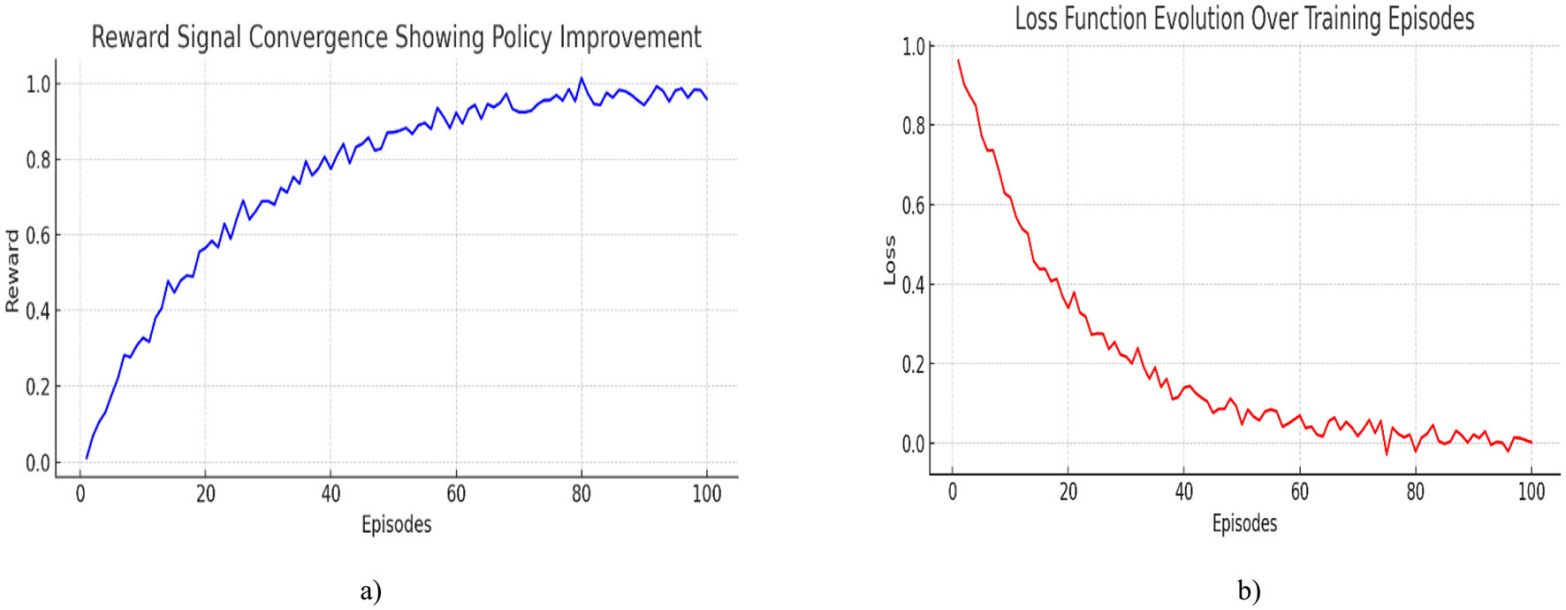
**(a)** Loss function evolution over training episodes **(b)** reward signal convergence showing policy improvement.

Figure 7a displays how the loss function evolves over 100 training episodes during the training of the Deep Q-Learning network. The loss represents the prediction error of the Q-network, how far off the predicted Q-values are from the target Q-values. As training progresses, the loss steadily decreases, which is a strong indicator that the model is learning effective value estimates for its state-action pairs. The exponential decay pattern with minor fluctuations shows convergence, suggesting that the model's predictions stabilize as it gets more experience.

Figure 7b displays the average reward per episode over the same training period. The reward is a measure of how successful the agent (DRL algorithm) is in achieving its goal, in this case, accurately predicting electric vehicle (EV) range. A steadily increasing reward trend indicates that the agent is improving its policy: it's making better predictions and decisions with each episode. The curve gradually plateaus, showing convergence to an optimal or near-optimal policy.

**Figure 8:**
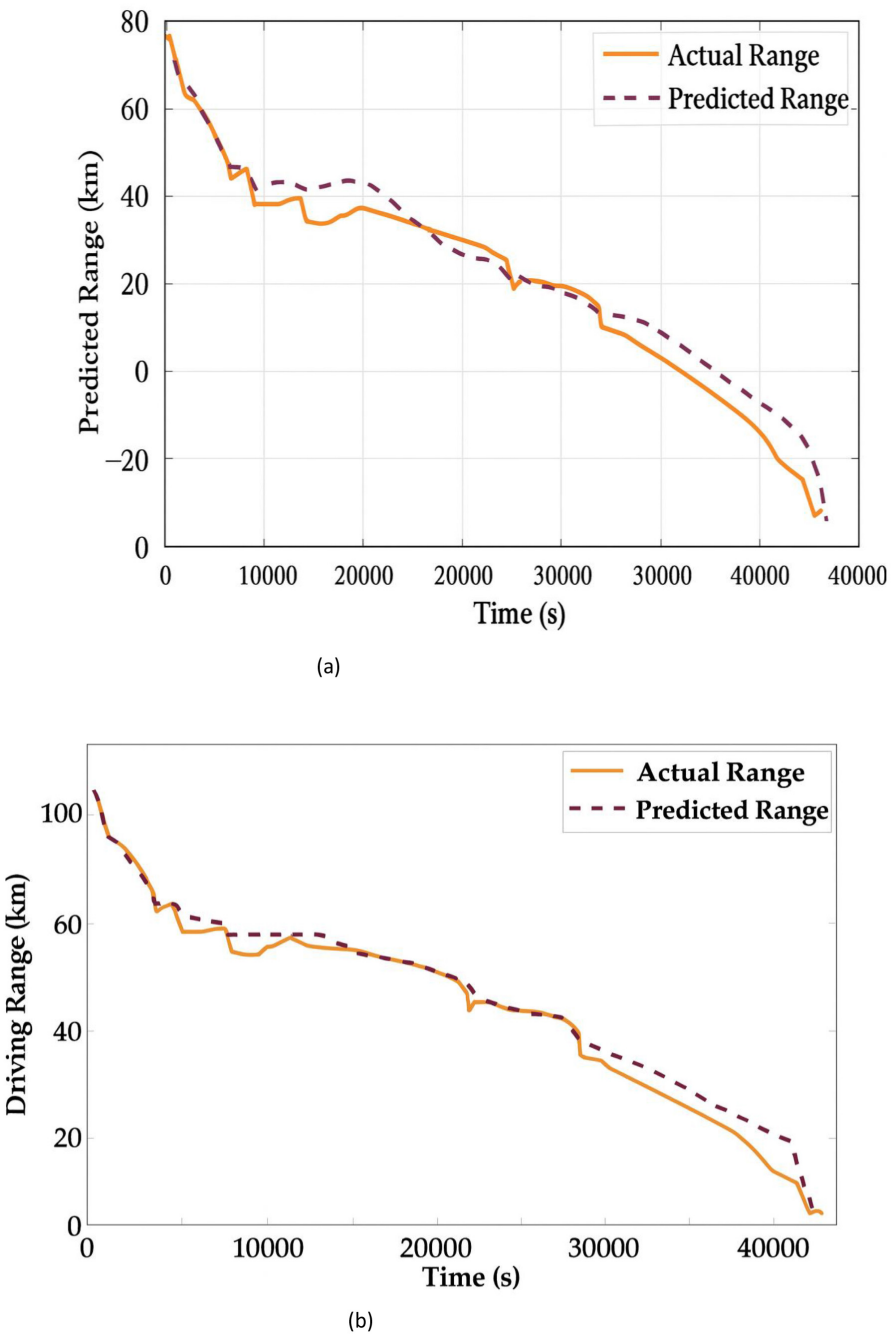
Predicted range of driving for **(a)** independent run and **(b)** 10-fold cross-validation.

The actual and predicted range by the proposed method for independent and 10-fold cross-validation is shown in Figure 8. From Figure 8a the average predicted error in an independent run is 0.0191 km and 0.0214 km for MAE and RMSE, respectively. In the case of 10-fold cross-validation (Figure 8b), the error is 0.0152 km and 0.02 km for MAE and RMSE, respectively.

**Figure 9:**
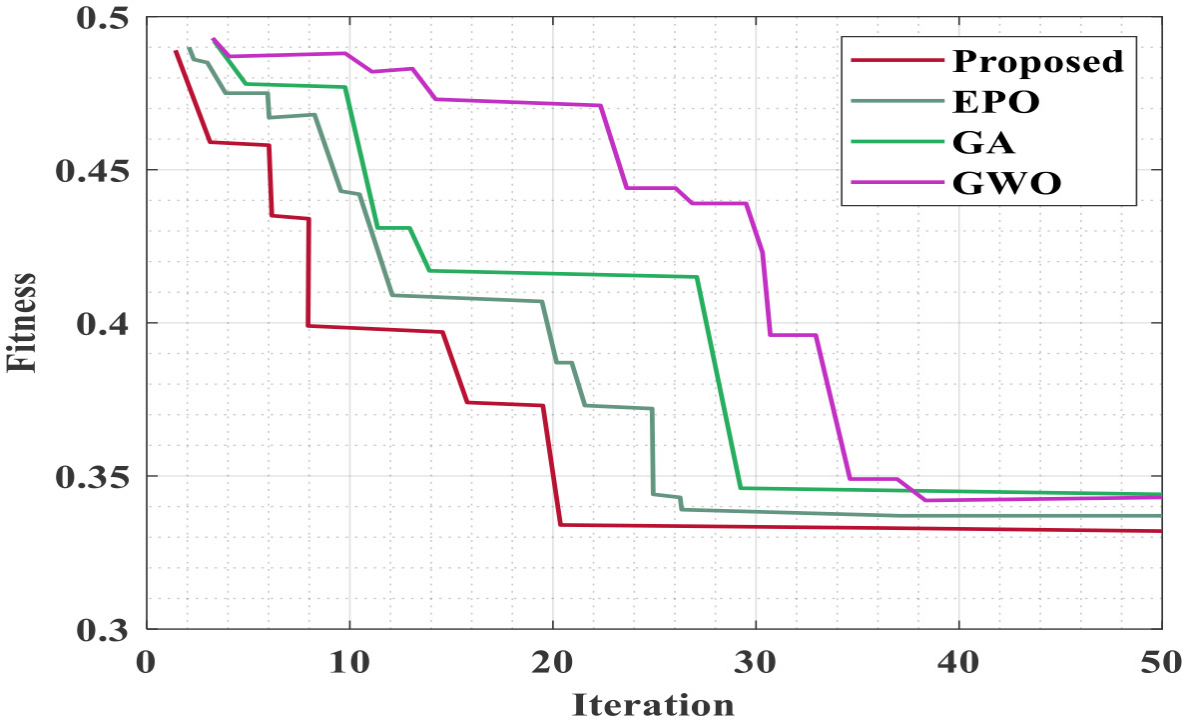
Convergence plot of the proposed optimization to reduce error in deep Q-learning.

The convergence of optimization is compared with existing algorithms such as emperor penguin optimization (EPO), genetic algorithm (GA), and gray wolf optimization (GWO), as shown in Figure 9. As a result, the proposed method has reached the global minimal value with the 20th iteration, whereas the other optimization takes 27, 29, and 38 iterations with high error values for EPO, GA, and GWO, respectively.

The comparison of the proposed method in contrast to state-of-the-art techniques is given in Tables 5, 6. In Table 3, the performance measures such as MAE and average range error are given. From the table, it is seen that the MAE is highly reduced for the machine learning method, as blended light gradient boosting regression tree and extreme gradient boosting regression tree have the value of 0.64, while the neural network method transformer architecture has 0.07. The error of those methods has been reduced by the proposed method in the range of 61.86% and 4.86% for machine learning and transformer architecture.

**Table 5:** MAE and error comparison of the proposed method with state-of-the-art techniques.

**References**	**Method**	**MAE (km)**	**Error (%)**
Sun et al. (2019)	Gradient boosting decision tree	0.678	[−1.41, 1.58]
Zhao et al. (2020)	Light gradient boosting regression tree	1.29	[−1.65, 1.65]
	Extreme gradient boosting regression tree	1.27	[−1.5, 1.5]
	Blended method	0.64	[−0.2, 1.2]
Wei et al. (2022)	Fuzzy c-means clustering-based online estimation	3	[0.94, 1.12]
Koohfar et al. (2023)	Recurrent neural network	0.427	-
	Long short-term memory	0.427	-
	Transformer architecture	0.07	-
Proposed	Deep Q-learning (independent run)	0.0191	[−0.28, 0. 4]
	Deep Q-learning (10-fold cross-validation)	0.0152	[−0.23, 0.34]

**Table 6:** Comparison of RMSE and MAPE with existing techniques.

**Author**	**RMSE (km)**	**MAPE (%)**
Zhang et al. (2020)	0.159	12.68%
Sun et al. (2019)	0.278	-
Zhao et al. (2020)	1.32	4.31%
	1.35	4.12%
	0.94	3.27%
Eagon et al. (2022)	0.057	-
Wei et al. (2022)	2.51	7.38%
Ullah et al. (2022)	2.230	9.6%
	2.042	9.8%
	2.202	9.9%
	2.281	10.1
	2.255	10.9
Alshammari and Chabaan (2023)	1.89	21.6%
Koohfar et al. (2023)	0.564	-
	0.522	-
	0.085	-
Proposed (independent run)	0.0214	2.36%
Proposed (10-fold cross-validation)	0.02	2.14%

The RMSE of the proposed method is 0.0214 km when compared with other methods; the value is highly reduced. The proposed method reduced the RMSE to 6.36% with a transformer architecture and 3.56% with two recurrent neural networks.

### 3.4 Comparison with state-of-the-art methods

To validate the effectiveness of the proposed DRL-based range prediction framework, we compare its performance with recent state-of-the-art (SOTA) methods reported in the literature and implemented on our dataset under the same conditions:

- **LSTM**: Recurrent model suitable for temporal battery features.
- **Transformer**: Deep attention-based model for sequence modeling.
- **XGBoost**: Gradient-boosted decision tree popular for tabular datasets.
- **LightGBM**: Lightweight, faster variant of XGBoost.
- **Baseline DQN:** Deep Q-learning without fuzzy clustering or optimization.

The comparison was based on MAE, RMSE, and R^2^ metrics.

In Table 7, the proposed method achieves the lowest values of error and the highest R^2^ compared to all tested SOTA models, which validates the effectiveness of the hybrid model in learning from dynamic and noisy EV datasets.

**Table 7:** Comparison with state-of-the-art methods (SOTA).

**Model**	**MAE (km)**	**RMSE (km)**	**R^2^ Score**	**Convergence epochs**
XGBoost	0.0428	0.0541	0.84	–
LightGBM	0.0386	0.0498	0.87	–
LSTM	0.0279	0.0312	0.91	210
Transformer	0.0247	0.0284	0.92	205
Baseline DQN	0.0391	0.0502	0.86	230
Proposed DRL Method	0.0152	0.0200	0.96	140

## 4 Managerial and societal implications

The framework for driving range prediction based on DRL that is proposed, will enhance the fleet manager, EV producer, or mobility service provider's capabilities for operation related decisions. Specifically, both logistics and ride-hailing services could enhance battery utilization and limit unscheduled charging stops, while extending the overall lifecycle of the vehicle by enhancing accuracy of the range estimate.

From the perspective of end-users, improved range estimates take away range anxiety and greatly encourage the adoption of EVs. Given that electric mobility is expanding rapidly on a global level, an energy management framework like the one proposed as part of the study supports energy conservation and the reduction of environmental impact. When there are good predictions, they also support smart grid applications such as efficiently meeting higher demand for charging stations. While the focus of the study is on range prediction for electric vehicles, the statistical and learning approaches relied upon, including fuzzy k-means clustering, DRL, and metaheuristic optimization, have wider applications to other reliability and decision-making problems with uncertainty. A similar framework could be adopted for businesses, for example, studying system degradation, maintenance optimization, and fault detection in energy storage, and manufacturing and transportation related systems. As necessary, adaptive learning combined with probabilistic modeling falls in quite well with the nature of reliability analysis that emphasizes uncertainty quantification and performance prediction. Similar statistical models have been applied to complex hybrid communication systems (Panić et al., 2023), further confirming that such methods are transferable across domains and suitable for multi-component reliability assessments and dynamic system optimization.

## 5 Conclusion

This paper proposed a hybrid deep reinforcement learning framework by incorporating fuzzy k-means clustering and Pathfinder optimization to improve the accuracy of electric vehicle range prediction. The proposed model provides not only strong computational performance but also practical value in grid-aware charging management, infrastructure design, and mitigation of driver range anxiety across renewable and fossil-based power systems. Relatively low prediction errors evidence the reliability of the model for dynamic operating conditions: the RMSE is 0.0214 km, while the MAE is 0.0191 km. Despite these encouraging results, several uncertainties persist. Real-world applications may face variations in charging energy sources, ambient temperature, and driving behavior that may actually influence prediction accuracy. Furthermore, Q-learning restricts scalability in continuous and high-dimensional state-action spaces, while reliance on a proprietary dataset further constrains full reproducibility.

Limitations in the current work will be overcome by future endeavors, including grid-level considerations such as renewable generation variability and emission intensity, scaled-up coordination at the fleet level, and more advanced reinforcement learning algorithms such as DDPG, TD3, and PPO for increased adaptability and generalizability. This, therefore, strengthens predictive robustness and convergence efficiency within the developed framework to further contribute toward dependable range estimation and sustainable mobility planning. Overall, this work has demonstrated how the most recent developments in data-driven and AI methodologies can bolster energy efficiency in transport systems to become aligned with increased innovation and more sustainable urban development.

## Data Availability

The original contributions presented in the study are included in the article/supplementary material, further inquiries can be directed to the corresponding author.

## Footnotes

1 Available online at: https://www.kaggle.com/code/cindynz/electric-vehicle-range-prediction/#7.-Use-Forward-Feature-Selection-to-pick-a-good-model.
